# Through the Looking Glass: Surveillance Following Colonoscopic Polypectomy of Malignant Polyps

**DOI:** 10.7759/cureus.38027

**Published:** 2023-04-23

**Authors:** Balaji Jayasankar, Dinesh Balasubramaniam, Kirolos Abdelsaid, Kyle Frowde, Emily Galloway, Mohamed Hassan

**Affiliations:** 1 Colorectal Surgery, Belfast Health and Social Care Trust, Belfast, GBR; 2 General Surgery, Maidstone and Tunbridge Wells NHS (National Health Service) Trust, Maidstone, GBR; 3 General Surgery, Maidstone and Tunbridge Wells NHS (National Health Service) Trust, Tunbridge Wells, GBR; 4 General Surgery, East Kent Hospitals University NHS (National Health Service) Foundation Trust, Canterbury, GBR; 5 Surgery, Maidstone and Tunbridge Wells NHS (National Health Service) Trust, Tunbridge Wells, GBR

**Keywords:** colorectal polyp, sessile serrated adenoma, pedunculated polyp, git endoscopy, surgical endoscopy, colon cancer and colon polyps, colonoscopy and polypectomy

## Abstract

Introduction

Colonoscopic polypectomy is a well-established screening and surveillance modality for malignant colorectal polyps. Following the detection of a malignant polyp, patients are either put on endoscopic surveillance or planned for a surgical procedure. We studied the outcome of colonoscopic excision of malignant polyps and their recurrence rates.

Methods

We performed a retrospective analysis over a period of five years (2015-2019) of patients who underwent colonoscopy and resection of malignant polyps. Size of polyp, follow-up with tumour markers, CT scan, and biopsy were considered individually for pedunculate and sessile polyps. We analysed the percentage of patients who underwent surgical resection, the percentage of patients who were managed conservatively, and the percentage of recurrence post-excision of malignant polyps.

Results

A total of 44 patients were included in the study. Of the 44 malignant polyps, most were present in the sigmoid colon at 43% (n=19), with the rectum containing 41% (n=18). The ascending colon accounted for 4.5% (n=2), transverse colonic polyps were 7% (n=3), and the descending colon polyps were 4.5% (n=2).

Pedunculated polyps made up 55% (n=24). These were Level 1-3 based on Haggits classification; 14 were Haggits Level 1, eight were Haggits Level 2, and two were Haggits Level 3. The rest were sessile polyps making up 45% (n=20). Based on the Kikuchi classification, these were predominantly SM1 (n=12) and SM2 (n=8).

Out of 44 cases, 11% (n=5) underwent surgical resection on follow-up in the form of bowel resection. This included three right hemicolectomies, one sigmoid colectomy, and one low anterior resection. Seven per cent (n=3) underwent endoscopic resection as trans-anal endoscopic mucosal resection (TEMS) and 82% (n=36) of the remaining cases were managed with regular follow-up and surveillance.

Conclusions

Colonoscopic polypectomy offers excellent benefits in detecting colorectal cancer and treating pre-malignant polyps. Colonoscopic polypectomy provides excellent benefits in colorectal cancer (CRC) detection and treatment of malignant polyps. However, it remains to be seen if post-polypectomy surveillance for low-risk polyp cancers would require a change in surveillance.

## Introduction

Colorectal cancer (CRC) represents a significant disease burden in the United Kingdom (UK), with around 40,000 new diagnoses yearly. A colonoscopy is a gold standard for the diagnosis of CRC [1,2].

Most CRCs develop from pre-malignant polyps. The majority of the polyps are slow-growing, often taking years to reach considerable size. Bowel cancer screening is crucial in preventing cancer development by removing pre-malignant polyps [1,3,4]. When endoscopic removal of polyps is inadequate or based on subsequent malignant pathology, some patients would require a formal bowel resection. The surveillance post-endoscopic resection of malignant polyps is not adequately supported with randomised controlled trials [2-5].

The National Comprehensive Cancer Network® (NCCN®), the British Society of Gastroenterology (BSG), and the Association of Coloproctology of Great Britain and Ireland (ACPGBI) provide explicit guidelines for surveillance post-cancer resection; however, they do not distinguish the follow-up between non-polyp colonic/rectal cancers from polyp cancers in the colon/rectum. Even though the efficacy of detection and diagnosis from colonoscopy is high, there is a lack of evidence to support the continuous post-polypectomy surveillance with colonoscopy [2-4].

Post-polypectomy surveillance places a severe burden on the system; it is estimated that around 150,000 endoscopies are done in the UK every year as surveillance endoscopies, the efficacy of which is questionable [3,5]. BSG has provided recommendations for follow-up post colon/rectal cancer resection and benign polyp excision. However, a follow-up pathway specifically for malignant polyps based on ‘pathology’ is lacking [1]. In our study, we aim to look at the outcomes of colonoscopic excision of malignant polyps.

## Materials and methods

This retrospective cohort study was conducted under Maidstone and Tunbridge Wells NHS (National Health Service) Trust, UK, which is a trust with district general hospitals in Kent, England, serving a population of about 760,000 people. 

Inclusion criteria

The study included all patients who underwent colonoscopic polypectomy between January 2015 to December 2019 (five years). All patients had a proven diagnosis of malignant polyp on histology. The polyps with low malignant potential following discussion at the multidisciplinary meeting were included.

Exclusion criteria

Patients who underwent colonoscopy with a cancer diagnosis requiring surgical resection following index procedure were excluded from the study. Malignant polyps with high potential were also excluded from the study. The multidisciplinary team did this as per national guidelines in the UK.

Process

A total of 44 patients who underwent polypectomy with histologically proven polyp cancer were included in the study. Following a discussion with the colorectal multidisciplinary team of surgeons, radiologists, pathologists, and oncologists, a decision was made for endoscopic surveillance. The patients were followed up for five years following the index colonoscopy and removal of the malignant polyp.

Follow-up included endoscopy (colonoscopy/sigmoidoscopy), carcinoembryonic antigen (CEA) levels, imaging (contrast CT/magnetic resonance imaging), and clinic appointments. The follow-up was at an interval of three, six, and nine months for the first year, and then six monthly till five years. Colorectal clinical nurse specialists conducted the follow-up as per our local guidelines. We had two local guidelines, one for colonic polyp cancers and another for rectal polyp cancers. The post-polypectomy follow-up structure for polyp cancers is given in Table 1.

**Table 1:** Post-polypectomy follow-up structure for polyp cancers CT: CT Abdomen and Pelvis; CT CAP: CT Chest, Abdomen, and Pelvis; MRI: MRI Rectum; Flexi Sig: Flexible Sigmoidoscopy; CEA: Serum Carcinoembryonic Antigen

Consultant				
Date of procedure				
	Test	Request	Date	Result
Colonic Polyps				
6 Months	CEA			
Face-to-face appointment				
1 Year	CEA			
	CT			
	Colonoscopy			
18 Months	CEA			
2 Years	CEA			
	CT			
2.5 Years	CEA			
3 Years	CEA			
4 Years	CEA			
	Colonoscopy			
5 Years	CEA			
	CT			
Rectal Polyps				
3 Month	Flexi Sig			
	MRI			
6 Month	CEA			
Face-to-face appointment	Flexi Sig			
	MRI			
9 Month	Flexi Sig			
	MRI			
1 Year	CEA			
Face-to-face appointment	Colonoscopy			
	MRI			
	CT CAP			
15 Month	Flexi Sig			
	MRI			
18 Month	CEA			
Telephone appointment	Flexi Sig			
	MRI			
21 month	Flexi Sig			
	MRI			
2 Year	CEA			
Face-to-face appointment	Flexi Sig			
	MRI			
	CT CAP			
2.5 Year	CEA			
Telephone appointment	Flexi Sig			
	MRI			
3 Year	CEA			
Telephone appointment	Flexi Sig			
	MRI			
3.5 Year	CEA			
Telephone appointment	Flexi Sig			
	MRI			
4 Year	CEA			
Telephone appointment	Flexi Sig			
	MRI			
4.5 Year	CEA			
Telephone appointment	Flexi Sig			
	MRI			
5 Year	CEA			
Face to face appointment	CT CAP			
	Colonoscopy			

Colonoscopy was carried out by the Joint Advisory Group (JAG) of GI endoscopists, accredited with key performance indicators fulfilling JAG criteria. The records were checked for the successful completion of the whole procedure. Pedunculated and sessile malignant polyps were classified based on Kikuchi or Haggits classification.

The primary outcome was to look at the successful follow-up of all patients following the endoscopic removal of malignant polyps. Our secondary outcome was the number of recurrences and the rate of surgical resection.

Data collection

Retrospective data were collected from the hospital records and anonymised. Colonoscopic data was obtained from the endoscopy reports, noting the size and location of polyps and the completeness of removal. These were correlated with the pathology reports tabulating the size, type of polyp, histology (Kikuchi and Haggits), completeness of removal, margins, and lymphovascular invasion. Follow-up data was taken from the patient records maintained by the colorectal care nurses.

Statistical analysis

Continuous variables were reported as median (range), and discrete variables were expressed as n (%) unless otherwise specified. Data analysis was conducted using IBM SPSS Statistics for Windows, Version 22.0 (Released 2013; IBM Corp., Armonk, New York, United States).

## Results

Patient characteristics

A total of 44 patients were included in the study. The majority of the participants (36%; n=16 ) were in the age group of 71-80 years; the least number of participants (18%; n=8) were in the age group of 51-60 years; 25% (n=11) were in the age group of 61-70 years and 20% (n=9) were in the age group of 81-90 years. The number of males (59%; n=26) was more with a male-to-female ratio of 1.4:1.

Polyp characteristics

A total of 56 polyps were detected, accounting for patients presenting with synchronous polyps. The non-cancerous synchronous polyps (n=12) were excluded from the analysis. These consisted of four cases of tubulovillous adenoma and three of tubular adenoma. Tubulovillous adenoma with dysplasia was found in three patients and two instances of hyperplastic polyps.

Site and size of polyps

Out of the 44 malignant polyps, most were present in the sigmoid colon (43%; n=19), with the rectum containing 41% (n=18). The ascending and descending colons accounted for 9% (n=2+2) each. Transverse colonic polyps were 7% (n=3). The predominant size of the resected polyps was >/=10mm, making up 66% (n=29) of the total resection. Of all other polyps, 34% (n=15) were <10mm in size. Amongst the ones >10mm, 19 polyps measured between 10-20mm, and 10 polyps were >20mm. 

Pathology of the polyps

All 44 polyps were proven malignancy with histologically confirmed adenocarcinoma. Further, the histology makeup was as follows: pedunculated polyps made up 55% (n=24). These were of Levels 1-3 based on Haggits classification. There were 14 of Haggits Level 1, eight of Haggits Level 2, and two of Haggits Level 3. The rest were sessile polyps making up 45% (n=20). Based on the Kikuchi classification, these were predominantly SM1 (n=12) and SM2 (n=8)

Outcome following polypectomy

All 44 cases were followed up in accordance with the post-polypectomy follow-up structure for polyp cancers (Table 1). There were no cases of missed follow-up on surveillance, and there was no recorded morbidity and no mortality during surveillance. Out of 44 cases, 11% (n=5) underwent surgical resection on follow-up in the form of bowel resection. This included three right hemicolectomies, one sigmoid colectomy and one anterior resection. The patient with sigmoid colectomy also had concurrent diverticulosis. One anterior resection was carried out six months following the initial diagnosis. The right hemicolectomies were taken up within one year after the index procedure on follow-up. 

Seven per cent (n=3) underwent endoscopic resection as trans-anal endoscopic mucosal resection (TEMS) or a repeat excision for clear pathological margins. All three cases were within the first six months after the initial procedure. Eighty-two per cent (n=36) of the remaining cases were managed with regular follow-up and surveillance. Out of these, one patient required further treatment for recurrence in the form of an anterior resection. The patient with a rectal tumour with incomplete excision of the polyp with high-risk features and had been advised and counselled regarding surgery following index colonoscopy; however, he opted not to have the surgery and was put on follow-up. The patient underwent surgery in the form of anterior resection after four years of follow-up as the tumour had recurred.

Characteristics of unresectable polyps

Of the five that went on for further resection, two focused on adenocarcinoma with lymphovascular involvement requiring right hemicolectomy. Another right hemicolectomy was carried out for a positive margin following repeat excision. One patient had concurrent diverticulosis, with a positive margin on primary excision and opted for sigmoid colectomy. One with multiple polyps in the rectum resulted in anterior resection as the margin was positive following primary excision. One patient with a recurrence was noted. This patient had a rectal tumour with invasion into the submucosa and had refused surgery following index excision; however, he had an anterior resection as he had a recurrence following four years of follow-up.

Follow-up on excised polyps

Patients were assigned to a pathway of stratified follow-up by the colorectal nurses. Five years of follow-up with serial colonoscopy, imaging, and biochemical CEA were done. Further, these patients were followed up with telephone/face-to-face clinic appointments. Out of 36 patients managed conservatively, there was one recurrence. No mortality was noted.

## Discussion

Endoscopic polypectomy has been proven to have good outcomes for colonic polyps. Further, it is essential to prevent colorectal cancers (CRC). The technique and safety of such endoscopic removal have seen dramatic improvements in the recent past. Additional methods like endoscopic mucosal resection (EMR) and endoscopic submucosal dissection (ESD) have been safely validated for removing large polyps [2,3].

In clinical practice, most colonic polyps are amenable to endoscopic polypectomy. The resection of these and further evaluation are necessary during the index procedure [3-6]. The completeness of the endoscopic procedure in our study was 100% compared to the national average of around 80% [5-7]. This was also because experienced endoscopists carried out the index and surveillance endoscopies at our institutions for the 44 cases.

In our study, 11% (n=5) of patients received radical resection; this was lower compared to a large trial by Moss et al. [8]. The number of successful endoscopic resections was 82%, comparable to the study published by Moss et al., where endoscopic resection was successful in 89.2% of the cases [8]. However, we concede that our numbers were small (n=44) compared to Moss et al.'s study. The patients who received further intervention in our study for polyp removal were 7% (n=3).

Tanaka et al. recommended a single follow-up within three years following polyp resection for adenomas [6]. Histologic grading and invasion would guide us on patients requiring further surgery. Histologic grading of pedunculated tumours (Haggits Level 0-3) and sessile tumours Kikuchi (SM1-2) predominantly required only endoscopic follow-up and resection. The other reported studies reiterated endoscopic removal of early tumours is effective with curative intent [9-13]. Endoscopic removal with clear oncological principles laid the key to effective surveillance [14-16].

Our study highlights the necessity of surveillance following endoscopic polypectomy for polyp cancers. However, given the low level of recurrence, it begets the question if the surveillance employed is too frequent or too much. National Institute for Health and Care Excellence (NICE) recommended post-colonoscopic surveillance at two to six months following index polypectomy and six months after. It is important to note that this was amended in September 2022 after this study had taken place [1,9]. However, this helps to validate the need for a study such as ours.

Further guidelines and recommendations are required to formulate definitive surveillance intervals. The last recommendation was in 2013 [9]; hence, it is time to rethink our surveillance strategy. Our study also highlights that a cohort of patients with good prognostic factors can be put safely on surveillance with good multi-disciplinary team input. This will lead to a practice of organ preservation in colorectal cancers, thus having a positive impact on patient outcomes and services [15].

The data on patient outcome is unclear following adherence to longitudinal follow-up [11,8]. Our study noted no mortality in our five-year follow-up with no apparent morbidity to patients. Some studies have shown that regular follow-up after index colonoscopy offers additional benefits in detecting CRC and missed polyps [12-14]. However, it is yet to be determined if these benefits would be the same for a low-risk category.

We acknowledge that our study has its limitations. Primarily it is a relatively small study in terms of patient numbers, and a more robust study will help to ascertain the importance of such follow-ups. Our analysis also does not detail the histopathological diagnosis of polyp cancers. The study looks at the follow-up pathway of proven polyp cancers, which had been decided following multidisciplinary team input. The pathological features of the polyps were out of the scope of this study; the inclusion of these will help to make a reasoned call on the existing pathways.

## Conclusions

Colonoscopic polypectomy provides excellent benefits in CRC detection and treatment of malignant polyps. However, it remains to be seen if post-polypectomy surveillance for low-risk polyp cancers would require a change in surveillance. Frequent endoscopies place a heavy toll on the system, and high-powered randomised controlled studies might offer more precise answers. More research and explicit guidelines are required for post-polypectomy surveillance in polyp cancers.
